# Voltage-dependent Ca^2+^ channels promote branching morphogenesis of salivary glands by patterning differential growth

**DOI:** 10.1038/s41598-018-25957-w

**Published:** 2018-05-15

**Authors:** J. M. Kim, S. Choi, S. W. Lee, K. Park

**Affiliations:** 1 Department of Dentistry, CHA Bundang Medical Center, CHA University, Seongnam, 13496 South Korea; 2 Department of Physiology, School of Dentistry, Seoul National University and Dental Research Institute, Seoul, 03080 South Korea

## Abstract

Branching morphogenesis is a crucial part of early developmental processes in diverse organs, but the detailed mechanism of this morphogenic event remains to be elucidated. Here we introduce an unknown mechanism leading to branching morphogenesis using mouse embryonic organotypic cultures with time-lapse live imaging. We found spatially expressed L-type voltage-dependent Ca^2+^ channels (VDCCs) in the peripheral layers of developing epithelial buds and identified the VDCCs as a core signaling mediator for patterning branching architecture. In this process, differential growth in peripheral layers by VDCC-induced ERK activity promoted cleft formation through an epithelial buckling-folding mechanism. Our findings reveal an unexpected role of VDCCs in developmental processes, and address a fundamental question regarding the initial process of branching morphogenesis.

## Introduction

Branching morphogenesis is an essential developmental process in early organogenesis of diverse organs such as the lungs, kidneys, and many types of glands^1^. Branching morphogenesis increases material transport efficiency by expanding the surface area within the confined organ space, and organizes the organ primordia into a functional complex through reciprocal interactions between the epithelium and surrounding mesenchyme^2,3^. In this process, the epithelial bud presents a characteristic morphological pattern depending on the organ type though there is a largely shared developing mechanism. An epithelial bud of the salivary gland is spatially divided by cleft formation for branching, while outward growth of the epithelial tube is pronounced in the lung; however, epithelial proliferation is essentially required for ordered developmental process in both cases^1,4,5^. To date, a number of extra/intracellular components involved in this genetic program have been introduced. Extracellular matrix fibronectin and the intracellular transcription regulator Btbd7 are systemically involved in branch propagation by regulating E-cadherin expression in lung and salivary gland cultures, and extracellular signal-related kinase (ERK) activity is an essential regulator of the shape and direction of lung epithelial tubes^6–8^. Importantly, growth factors were known as inductive signals for guiding the branching patterns in a spatiotemporal manner^9^. Despite such plentiful information, an accurate mechanism and related key signaling mediators underlying initiating and patterning of the branching process have not yet been clearly identified.

The voltage-dependent Ca^2+^ channel (VDCC) is a protein complex that mediates Ca^2+^ entry upon changes in the membrane potential of excitable cells. VDCCs regulate a variety of cellular events, such as actomyosin contraction, synaptic transmission, and hormonal secretion according to the interacting partners with entered Ca^2+ ^^10^. In addition to these canonical functions, VDCCs are involved in the other cellular functions including cell motility, front-rear polarity, and immune response, which are mainly studied in non-excitable cell types^11–13^. Notably, the expression of several subtypes of VDCCs was reported in the kidneys and developing lungs^14,15^. These evidences reflect the unconventional functional aspects of VDCCs in non-excitable biological contexts, including the epithelial organ development, and it is possible that these processes might be governed by a different mechanism from that of excitable cells.

Here, we introduce the critical role of a voltage-dependent calcium channel (VDCC) in the initial phase of branching morphogenesis. Using various bioimaging techniques, we revealed that localized VDCC activity establishes differential growth patterns in developing epithelial buds, resulting in the spatial rearrangement of branching structures.

## Results

### The effect of L-type voltage-dependent Ca^2+^ channels (VDCCs) on branching morphogenesis

For morphological studies, we utilized an *ex vivo* model of the mouse embryonic submandibular gland (SMG), which gives a clear and reproducible morphological read out during the early developmental period^16^. Using this model, we sought to identify an unknown inductive signaling component for branching morphogenesis. We focused on extracellular Ca^2+^ ([Ca^2+^]_o_) as a possible candidate based on a previous report describing the involvement of Ca^2+^ influx in craniofacial development, which partly shares a common developmental origin and mechanism with the salivary glands^17^.

**Figure 1:**
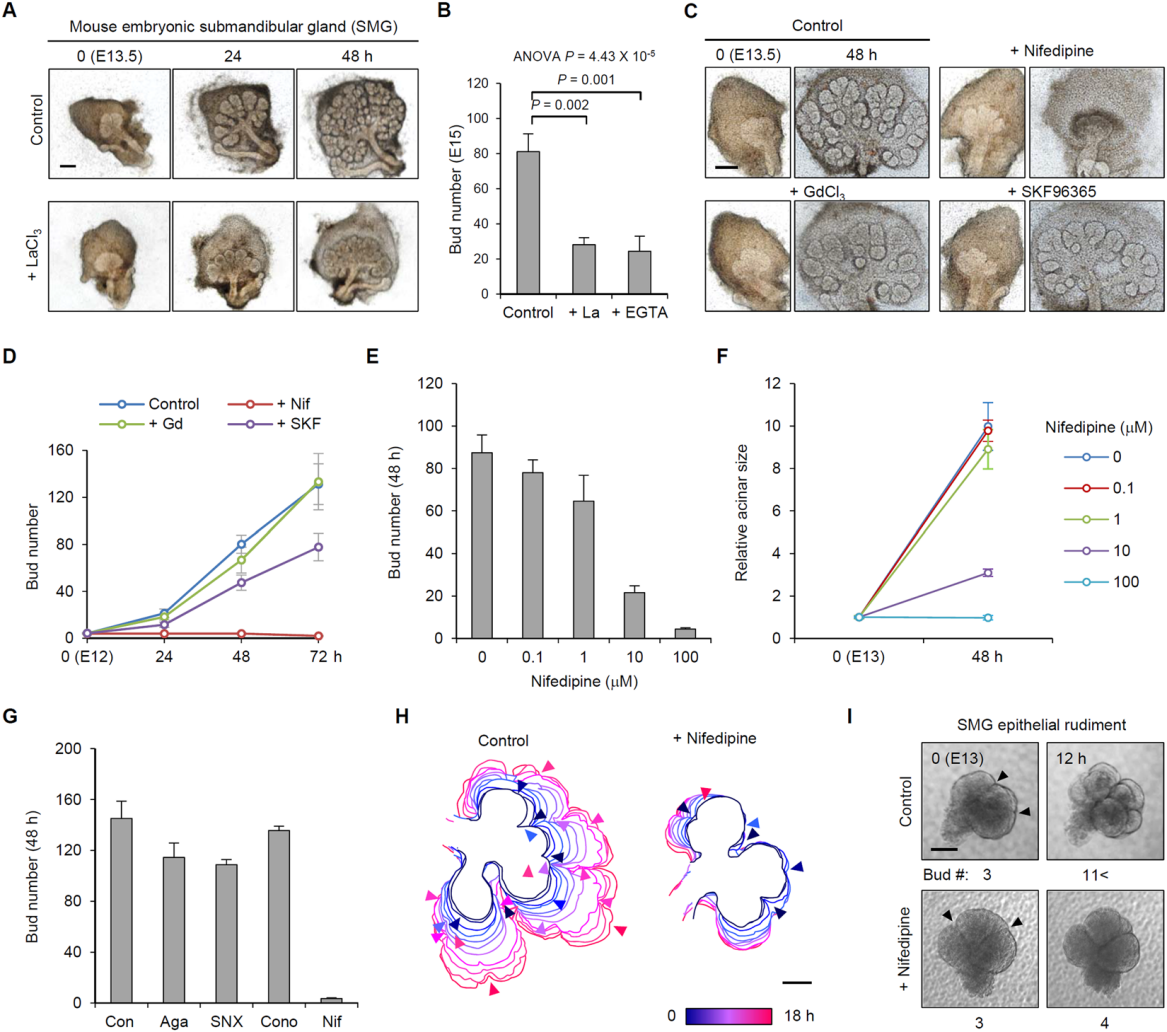
The effect of L-type voltage-dependent Ca^2+^ channels (VDCCs) on branching morphogenesis. (**A**) Morphological changes of SMG cultures (E13.5) upon 500 μM LaCl_3_ treatment. (**B**) Bud numbers of SMG cultures upon 500 μM LaCl_3_ (La) and 1 M EGTA treatment. n = 7, Data are represented as mean ± SEM. (**C**) Representative images of SMG cultures treated with various Ca^2+^ channel inhibitors. (**D**) Bud numbers of SMG cultures (E12) upon treatment with various Ca^2+^ channel inhibitors. Nif: 100 μM nifedipine; Gd: 500 μM GdCl3; SKF: 10 μM SKF 96365, n = 7, Data are represented as mean ±SEM. (**E**) Bud numbers of SMG cultures (E13) upon different concentrations of nifedipine treatment for 48 h. n = 5. Data are represented as mean ± SEM. (**F**) Relative acinar size of SMGs (E13) upon different concentrations of nifedipine treatment. n = 5. Data are represented as mean ±SEM. (**G**) Epithelial bud numbers of SMGs (E13.5) upon treatment with antagonists for different types of VDCCs: 2 μM w-Agatoxin IVA (Aga, P-type); 2 μM SNX 482 (SNX, R-type); 10 μM w-Conotoxin GVIA (Cono, N-type). n = 6. Data are represented as mean ±SEM. (**H**) Time-course changes of bud outline of developing SMG cultures. Arrowheads indicate the cleft initiation points. (**I**) Time-lapse images of epithelial rudiment cultures (E13) upon 100 μM nifedipine treatment. Arrowheads indicate cleft sites. Panels below indicate the bud numbers. Scale bars: 200 (**A**,**C**), 100 μm (**H**,**I**).

We first treated SMG cultures with LaCl_3_ (a general Ca^2+^ channel inhibitor) to investigate the role of [Ca^2+^]_o_ in branching morphogenesis. Notably, LaCl_3_-treated SMG cultures showed immature development patterns, as represented by a 65.3% reduction in the number of epithelial buds (Fig. 1A,B). The importance of [Ca^2+^]_o_ in branching morphogenesis was supported by a similar pattern of SMG morphology following chelation of [Ca^2+^]_o_ by ethylene glycol-bis(β-aminoethyl ether)-N,N,N′,N′-tetraacetic acid (EGTA) (Fig. 1B). We next searched for a possible mediator that links [Ca^2+^]_o_ and branching morphogenesis using a database of salivary gland gene expression (http://sgmap.nidcr.nih.gov/sgmap/sgexp.html)^18^. Among the molecules related to [Ca^2+^]_o_ transport, we identified that several types of VDCCs, transient receptor potential (TRP) channels, and stromal interaction molecule (STIM) 1 are highly expressed in the critical period for branching organization [embryonic day (E) 12–16]. We then blocked the action of each of these components in developing SMG cultures with various chemical antagonists and found that nifedipine (an L-type VDCC antagonist) strikingly diminished new bud formation (Fig. 1C,D). We then confirmed the dose dependency and L-type specificity of this inhibitory effect (Fig. 1E–G). Nifedipine also clearly suppressed branching morphogenesis in mouse embryonic lung cultures, suggesting that this finding has broad implications for diverse organs (Supplementary Fig. S1A–C). To accurately evaluate the morphological consequences, we monitored developing SMGs for 18 h (from E13) by time-lapse live imaging. The serial images of the development pattern revealed that nifedipine-treated SMGs failed to progress a new cleft, resulting in no additional bud formation (Fig. 1H and Supplementary Video 1). We next cultured isolated epithelial buds of SMGs (eSMGs) and verified the purity of the cultures (Supplementary Fig. S1D,E) and the inhibitory effect of nifedipine on cleft formation (Fig. 1I). These results indicate that a major driving force of cleft formation is derived from the intrinsic physiological effect of VDCCs in the epithelial bud and not in the surrounding mesenchyme.

### Localized expression of VDCCs in developing SMGs

**Figure 2:**
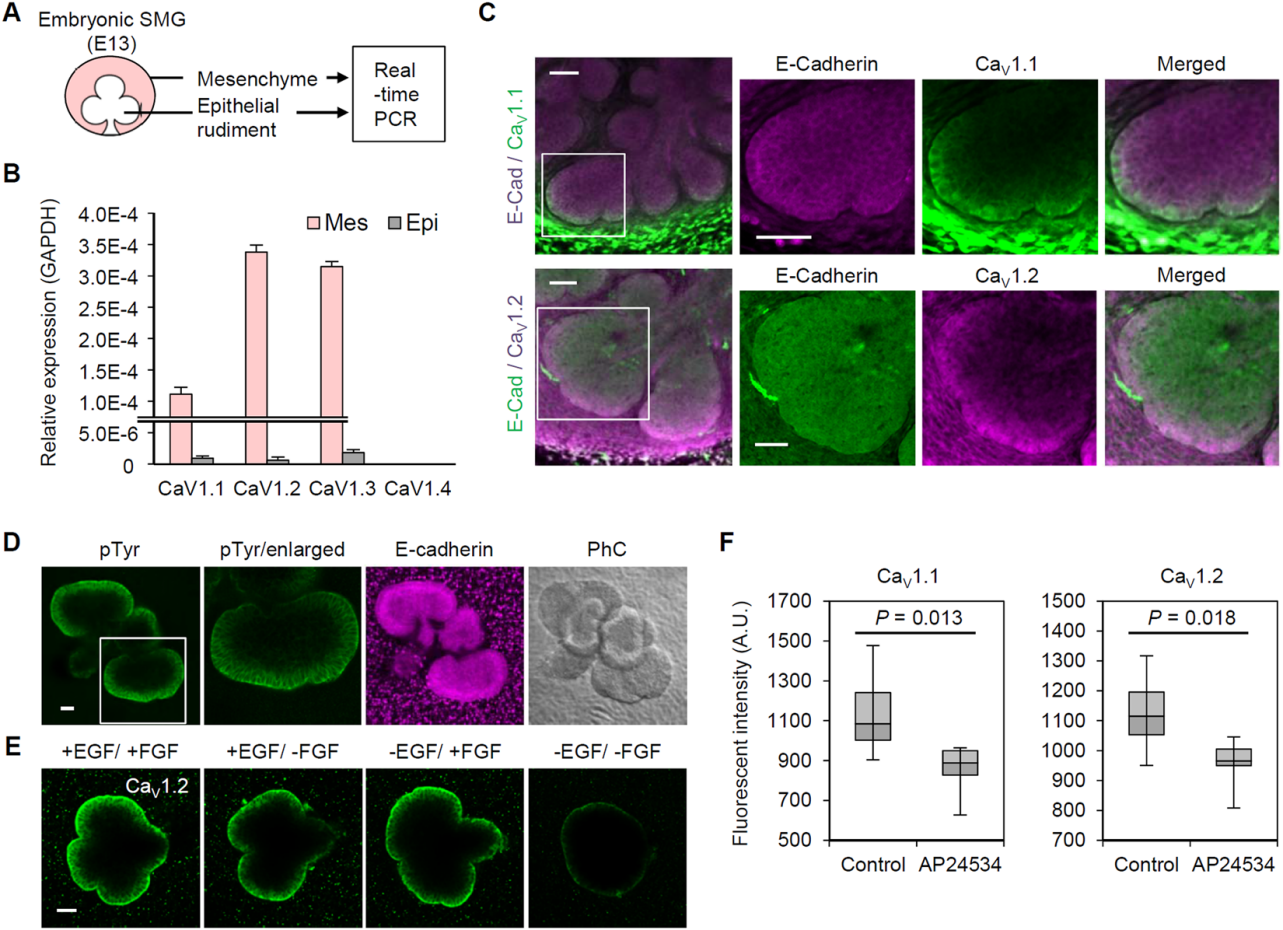
Localized expression of VDCC in developing SMGs. (**A**) Experimental design for quantitative real-time PCR of isolated SMG cultures. (**B**) mRNA expression profile of VDCC subtypes (Ca_V_1.1-1.4) in isolated SMG cultures. (**C**) Immunofluorescence images of SMG cultures labeled with VDCC (Ca_V_1.1 and 1.2) and E-cadherin. White boxes indicate the area enlarged in the right three panels. (**D**) Immunofluorescence images of SMG epithelial cultures (eSMGs) labeled with phosphorylated tyrosine (pTyr) and E-Cadherin. White boxes indicate enlarged area for right three panels. PhC: phase contrast. (**E**) Immunofluorescence images of VDCC-labeled eSMGs in growth factor-depleted culture media (6 h). (**F**) Relative intensity of immune-labeled VDCC subtypes in isolated eSMGs upon 100 nM AP24534 treatment. n = 7 (control); 6 (AP24534). Data are represented as median ±min/max. Scale bars: 50 μm.

This newly identified function of L-type VDCCs in epithelial bud development led us to verify the expression of these channels in SMG compartments (Fig. 2A). Among the four subtypes of L-type VDCC (Ca_V_1.1 to 1.4), three types (Ca_V_1.1 to 1.3) were detected in both the mesenchyme and epithelial buds, but the epithelial portion had a mRNA expression level of approximately 1% compared to the mesenchyme (Fig. 2B). Instead, immunostaining revealed a localized expression pattern of VDCCs that was exclusively concentrated in the peripheral cell layers of the epithelial buds (Fig. 2C). Based on quantitative analysis, over 50% of the VDCCs were expressed within the three outermost layers of the epithelial buds (Supplementary Fig. S2A). The same expression patterns were confirmed in eSMG (Supplementary Fig. S2B) and lung cultures (Supplementary Fig. S2C) by immunostaining and fluorescence *in situ* hybridization (Supplementary Fig. S2D). This characteristic localized expression pattern may explain the inconsistency between the obvious function of VDCCs in bud formation and the low expression of the channels in epithelial buds (Figs 1F and 2B). Moreover, a higher Ca^2+^ level was detected in the peripheral cell membranes of eSMGs by expression of a membrane-tethered Ca^2+^ biosensor (GCaMP6s-CAAX), implying functional expression of the channels (Supplementary Fig. S2E).

Next, we probed the molecular mechanism underlying localized expression of VDCCs. The growth factor –receptor tyrosine kinase (RTK) pathway is a representative signaling cascade that plays versatile roles in branching morphogenesis^3,19^. The growth factor signal exogenously guides spatial patterns of organ architecture through interaction with the extracellular matrix^20^. Therefore, we investigated RTK activity in epithelial buds by visualizing the spatial pattern of immunolabeled phosphorylation of tyrosine residues (pTyr) in eSMG cultures and a found striking pattern of pTyr concentrated in the peripheral epithelial layers (Fig. 2D). Based on this result, we determined that the RTK signal is essential for VDCC expression regardless of growth factor subtype specificity as demonstrated by the decrease in VDCC expression caused by removing epidermal growth factor (EGF) and/or fibroblast growth factor (FGF) from the eSMG culture media (see Methods section; Fig. 2E). The expression level of VDCCs was also significantly decreased by treatment with a pan-RTK inhibitor (AP24534) (Fig. 2F).

### Spatial relationship between VDCCs and the MAPK pathway

**Figure 3:**
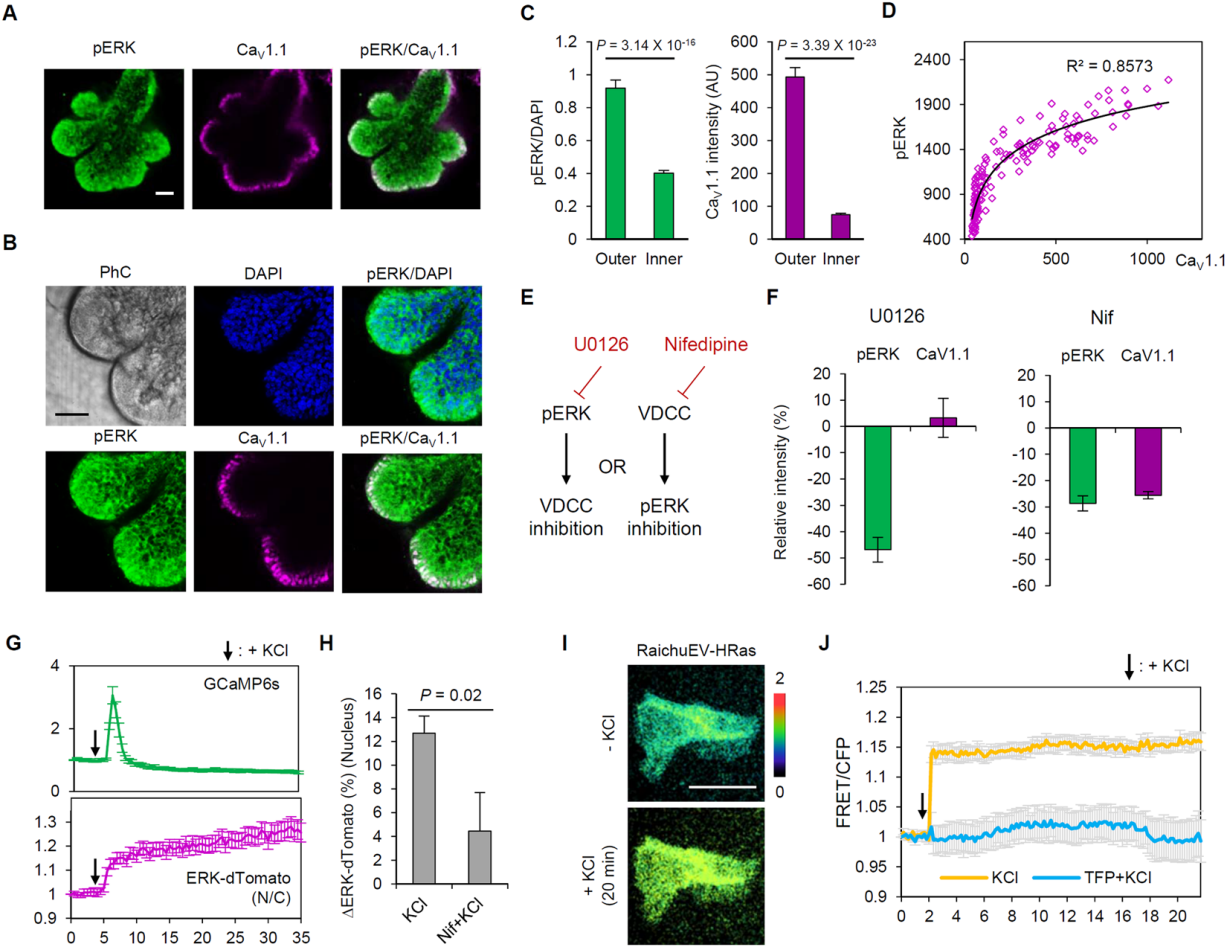
Spatial relationship between VDCC and ERK. (**A**) Immunofluorescence images of SMGs labeled with phosphorylated ERK (pERK) and Ca_V_1.1. (**B**) Enlarged images focusing individual buds of eSMGs. PhC: phase contrast. (**C**) Relative intensity of pERK/DAPI signals (left) and Ca_V_ 1.1(right) of epithelial cells in the outer and inner part of eSMGs. n = 72. Data are represented as mean ±SEM. AU: arbitrary unit. (**D**) Spatial correlation between the expression levels of Ca_V_1.1 and pERK signals in eSMG cultures. n = 144. (**E**) Experimental scheme for determining signaling hierarchy between ERK and VDCCs. (**F**) Intensity changes (%) in pERK and Ca_V_1.1 levels in the buds of SMGs cultures upon 10 μM U0126 (left, n = 16) and 100 μM nifedipine (right, n = 10) treatment. Data are represented as mean ± SEM. (**G**) Relative intensity of G-CaMP6s and ERK (nucleus/cytoplasm) signals in SMG-C6 cells. Arrows indicate the time point of 50 mM KCl treatment. n = 25. Data are represented as mean ± SEM. (**H**) Intensity changes (%) in nuclear ERK signals by 50 mM KCl with/without 100 μM nifedipine preincubation. n = 11. Data are represented as mean ± SEM. (**I**) Representative images of SMG-C6 cells expressing RaichuEV-HRas after 50 mM KCl treatment. (**J**) Relative changes in FRET/CFP signals induced by 50 mM KCl, upon 25 μM trifluoperazine (TFP) preincubation. n = 7. Data are represented as mean ±SEM. Scale bars: 50 μm.

Next, we searched for the signaling mediator of branching morphogenesis induced by localized VDCC activity. It has been reported that mitogen-activated protein kinase (MAPK) also shows localized activity confined to the peripheral region of the epithelial bud^21^, suggesting its correlation with VDCC expression patterns. Moreover, VDCCs are known to activate Ras, an upstream component of the MAPK pathway, through localized Ca^2+^–calmodulin (CaM) interaction^22,23^. Immunostaining results confirmed higher phosphorylated ERK (pERK) signals in the peripheral region of eSMG cultures (Fig. 3A,B), which were highly spatially correlated with VDCC expression patterns (Fig. 3C,D; R^2^ = 0.8573). To verify the signaling hierarchy between VDCCs and ERK, we treated SMG cultures with either U0126 (a MEK inhibitor) or nifedipine, and compared the resulting changes in respective signaling activity (Fig. 3E). While U0126 did not affect the expression level of VDCCs, nifedipine reduced ERK phosphorylation (−28.61%, Fig. 3F and Supplementary Fig. S3A), indicating that VDCCs are an upstream mediator of ERK. This hierarchy was additionally confirmed by simultaneous monitoring of intracellular Ca^2+^ (G-CaMP6s) and ERK activity (ERK-dTomato) in rat submandibular gland epithelial cells (SMG-C6) upon KCl depolarization. Application of KCl immediately increased G-CaMP6s signals, and subsequent nuclear translocation of ERK-dTomato was detected (Fig. 3G and Supplementary Video 2). This effect was significantly blocked by nifedipine treatment (Fig. 3H). We also dissected the signaling pathway that couples VDCCs to ERK, seeking to identify pathway intermediates. To this end, we conducted an in-depth study of Ras activity using fluorescence resonance energy transfer (FRET) probes (RaichuEV-HRas)^24^ in SMG-C6 cells (Fig. 3I). The activation of VDCCs induced a rapid and sustained increase in Ras activity, and this increase was completely abolished by preincubation with the Ca^2+^-CaM binding inhibitor, trifluoperazine (Fig. 3J). Taken together, these results clearly establish a connection between VDCC activity and ERK phosphorylation, demonstrating an intermediary role for Ca^2+^/CaM-dependent Ras activation. Because the Ras–MAPK pathway is also known as a downstream of RTKs, we next compared ERK activity in response to VDCC and growth factor signaling inputs through immunoblotting. KCl treatment yielded a higher pERK level in SMG-C6 cells than EGF treatment, and combined EGF-KCl treatment resulted in a synergistic increase in the phosphorylation level (Supplementary Fig. S3B). We then evaluated SMG morphology following U0126 application and confirmed a similar inhibitory effect with nifedipine treatment (Supplementary Fig. S3C,D). These data indicate that the VDCC–ERK cascade promotes branching morphogenesis in developing SMGs.

### Differential growth promotes cleft formation

**Figure 4:**
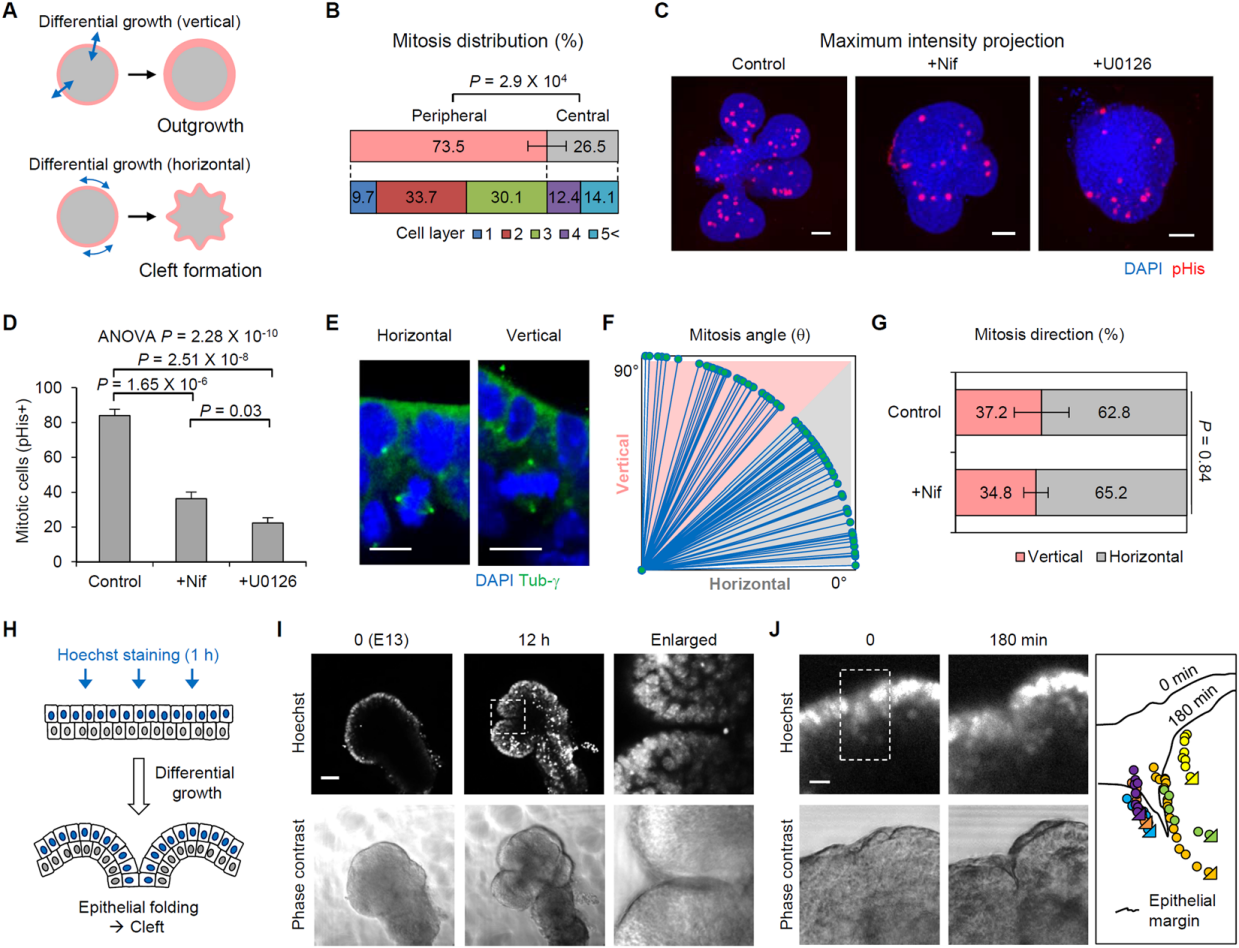
Differential growth promotes cleft initiation. (**A**) The determination of morphological patterns of epithelial buds by the orientation of differential growth. (**B**) Spatial distribution of mitotic cells in eSMGs. n = 7 buds. (**C**) Maximum intensity projection images (Z-axis) of mitotic cells in eSMGs treated with 100 μM nifedipine or 10 μM U0126. pHis: phospho Histone H3. (**D**) Mitotic cell numbers (pHis positive cells) in developing eSMGs. n = 7 (**E**) Representative images of the cells under horizontal (left) and vertical (right) mitosis. (**F**) Distribution of mitosis angle in developing eSMGs. n = 82 cells (**G**) Distribution of mitosis direction in developing eSMGs. n = 12 (control) and 15 (+100 μM Nifedipine) buds. (**H**) Experimental scheme for live imaging of developing epithelial bud with selective staining of the peripheral nucleus (see Methods section). (**I**) Epithelial dynamics (Hoechst-stained; upper panels) and phase contrast images (lower panels) of a developing eSMG. Dotted region indicates the epithelial folding site. (**J**) Tracking images of the movement of the peripheral nucleus. Right: tracking results of a single cell in the dotted region. Tirangular points in the tracking results indicate the end position of the nucleus. Data are represented as mean ± SEM. Scale bars: 10 (**E** and **J**), 50 μm (**C** and **I**).

How can VDCC–ERK signals trigger the branching process? We focused on the concept of differential growth, in which localized (or patterned) proliferation organizes epithelial architecture during the initial developmental process^25^. Given this background, we hypothesized that ERK-induced localized proliferation in the peripheral layers governs both bud outgrowth (increasing organ size) and cleft formation (increasing bud number), and that the fate of the developing pattern is determined by the mitosis orientation (Fig. 4A). In particular, an increase in peripheral cell density by differential growth with horizontally-directed mitosis was assumed to be a major driving force in cleft formation through epithelial buckling-folding mechanisms^26^. We initially quantified the local distribution of mitotic cells in branching epithelial buds to characterize the differential growth patterns (see Methods section; Supplementary Fig. S4A). As expected, 73.5% of mitoses occurred in the peripheral layers (defined as the outermost three layers) (Fig. 4B), and mitotic cell density in the developing buds was significantly reduced by nifedipine or U0126 treatment [84.00 (control), 36.28 (nifedipine), and 22.28 (U0126) mitotic cells; Fig. 4C,D). Next, mitosis orientation was measured based on the angle between the mitotic axis and the bud surface (Fig. 4E; see Methods section). The measured mitosis angle (θ) in the peripheral layers showed a higher distribution in the horizontal direction (0° < θ < 45°) than in the vertical direction (45° < θ < 90°) with an approximately 2:1 ratio [62.8% (horizontal) versus 37.2% (vertical); Fig. 4F,G]. However, inhibition of VDCCs did not result in a notable change in the mitosis orientation (Fig. 4G). In the U0126-treated buds, it was difficult to measure the mitotic angle due to lower mitotic cell density than in the nifedipine-treated group (Fig. 4D). These data indicate that the VDCC-ERK cascade is involved in inducing mitotic signals rather than in regulating mitotic orientation.

**Figure 5:**
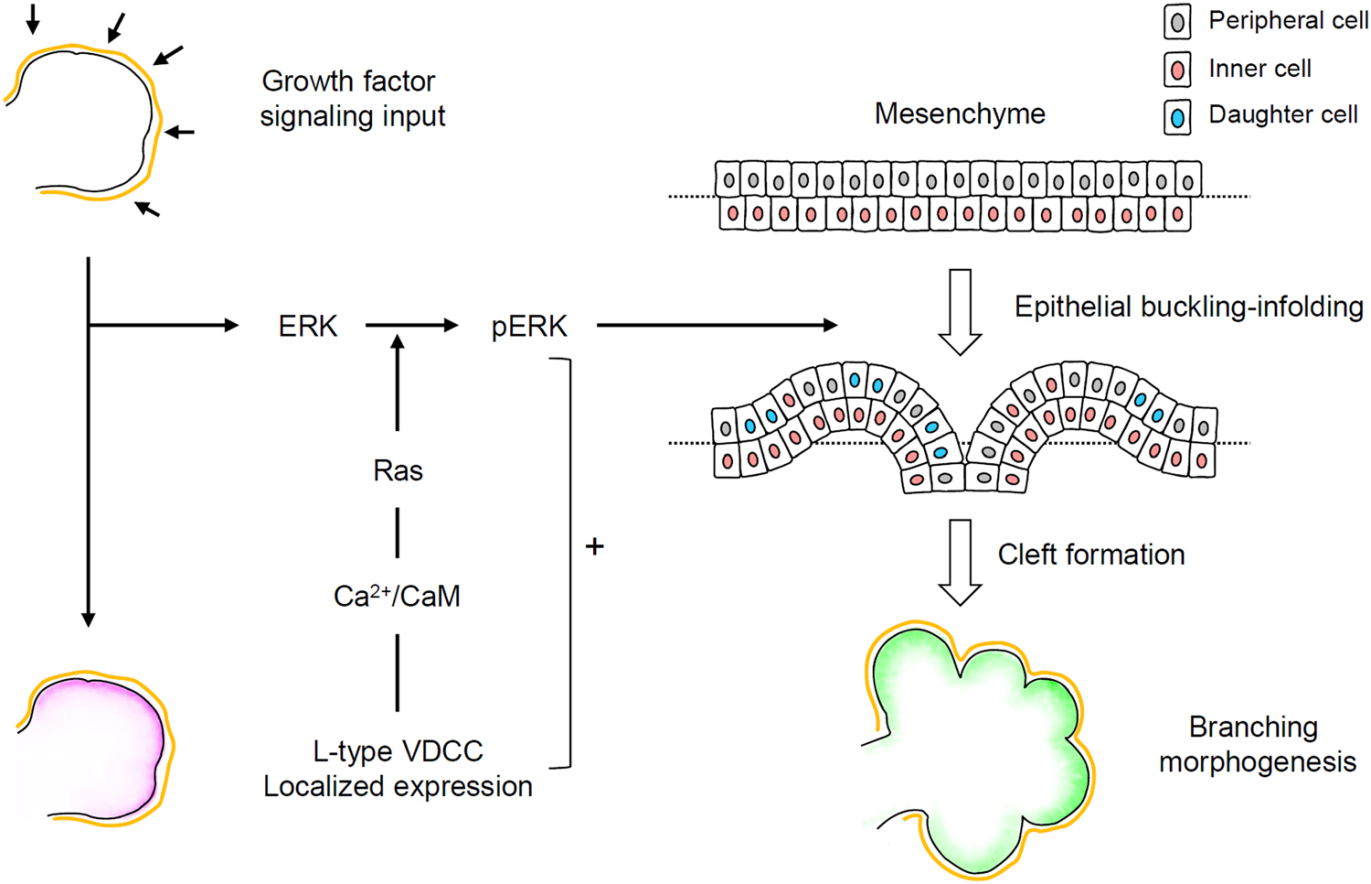
Schematic representation displaying the role of L-type VDCCs in branching morphogenesis. Localized expression of L-type VDCCs patterned by growth factor signaling input synergistically induces ERK phosphorylation. The differential growth of epithelial buds elicits spatial rearrangement of the peripheral cells, resulting in cleft formation through an epithelial buckling-folding mechanism.

We also investigated the spatial rearrangement of the peripheral epithelium of developing buds by live staining with Hoechst dye for a short period (<1 h), enabling selective staining of the peripheral nuclei (see Methods section; Fig. 4H). During a 12 h period (from E13), we confirmed the presence of epithelial folding at the cleft initiation site, demonstrated by the arrangement of stained epithelial nuclei along the cleft (Fig. 4I and Supplementary Video 3). High magnification time-lapse images over 3 h also revealed the inward movement of peripheral cells toward the cleft-forming direction (Fig. 4J; Supplementary Video 4). During the cleft-initiation process, we observed a gradual increase in the cell number in the peripheral layer along with an increase in the epithelial margin length (Supplementary Fig. S4B), indicating that increased peripheral cell density was a major triggering factor for spatial rearrangement of the epithelial structures. The increase in peripheral cell density was accelerated by outward migration of the inner cells, as characterized by pseudostratified-like epithelial regions in the outermost cell layer observed in fixed eSMG cultures (Supplementary Fig. S4C,D). These results support our hypothesis explaining the triggering mechanism of branching morphogenesis: localized epithelial proliferation is a crucial mechanism for epithelial infolding and the resultant cleft formation (Fig. 5).

## Discussion

In this study, we demonstrated that spatial rearrangement of epithelial layers by VDCC-induced localized proliferation is a key inducer of branching morphogenesis (Fig. 5). Epithelial proliferation is generally thought to play a central role in the overall developmental process by providing new cells to occupy the enlarging organ space. However, the functional relationship between epithelial proliferation and the branching process (including budding and clefting) has been a contentious issue in various epithelial organs^25,27^. Nakanishi *et al*. reported that proliferation of epithelial buds is not required for early-cleft formation in developing SMG cultures, a result at odds with our findings^28^. There are a number of possible explanations for these different outcomes. The first is limitations in the methods applied in both studies. The previous report used a DNA-based approach with X-ray irradiation and radioactive nucleotides. In our study, we blocked proliferation through chemical perturbation of MEK signaling and analyzed the proliferation rate by counting the mitotic cell number. Unfortunately, no method can ensure complete inhibition or accurate detection of cell proliferation, both of which are essential prerequisites for addressing questions relating to such a complex process. Second, the selection criteria for clefts in our study were different. As a practical matter, static images of developing buds are a somewhat ambiguous tool for detecting actual clefts, and thus we used a real-time monitoring system to accurately detect the whole process of the cleft formation (Fig. 1H,I). Using this method, we could exclude dimple-like structures, which occur through transient flexion of the outer epithelial layers. Overall, we suggest that these conflicts primarily reflect the different experimental approaches and interpretation of the data. Although previous reports have tended to regard epithelial bud proliferation as a phenomenon distinct from cleft formation, our work compels the conclusion that these two events are reciprocally related during early branching morphogenesis.

The effects of VDCC on branching morphogenesis seen in SMG cultures were experimentally reproduced in lung cultures (Supplementary Fig. S1A–C), enhancing the biological relevance of our findings. The ERK signal, which we determined acts as a core downstream effector of the branching process, was previously reported to regulate the length and thickness of developing lung branches by affecting mitosis orientation^8^. The mitosis angle was typically arranged toward the elongating direction of the airway tubes, and enhanced ERK activity perturbed this orientation, resulting in the alteration of branching patterns in developing lungs (reduced length and increased thickness). In SMG cultures, mitosis orientation was horizontally arranged in relation to the outer surface of epithelial buds, which might be the reason for the spherical shape of SMG buds rather than an elongated morphology. In this context, we found that ERK activity was preferentially involved in localized induction of mitosis rather than affecting orientation and that the spatial distribution of epithelial proliferation is crucial for patterning differential growth. Given this set of results, ERK activity and related mitotic characteristics-orientation and spatial distribution-can be regarded as crucial factors for determining branching patterns among different epithelial organs.

Moreover, we suggested the growth factor signal as a determinant factor of VDCC expression patterns. To date, diverse growth factors and related feedback systems have been introduced to account for the patterning of branching structures by computational modeling^29^. Recently reported model based on FGF-SHH feedback signals (ligand–receptor-based Turing mechanism) could explain a general mechanism for the regulation of stereotyped branching in diverse organs^30^. Through this study, we revealed that the growth factor signals patterning branching structures are also involved in patterning VDCC expression (Fig. 2D,F). Given signaling connectivity proposes that VDCC is a pivotal mediator in the ligand–receptor-based developmental program by providing supporting proliferation signals.

This report not only provides a plausible explanation for the mechanism of branching morphogenesis, also expands the functional range of VDCCs beyond the previously well-known functions in excitable cells such as synaptic transmission and cardiac pacemaking^31^. We also introduced the synergistic role of VDCCs and growth factor signals in ERK activation, which is crucial for the proliferation of epithelial organs. The VDCC-ERK signaling cascade was firstly introduced in depolarization-induced ERK activation in neurons, which is crucial for synaptic plasticity and learning and memory^22,32^. In that context, Ca^2+^ influx through VDCC transduces signals from plasma membrane to nucleus through CaM-dependent MAPK pathway^23^. In this study, we demonstrated the spatial relationship between VDCC expression and ERK activity, as well as the connectivity of the signals, using SMG culture models and cell lines expressing diverse genetically-encoded biosensors (Fig. 3G–J). However, the complete map of this pathway has not been established, and specifically, the identity of the guanine nucleotide exchange factor (GEF) responsible for CaM and Ras activation remains an important question. In light of our results, we expect to elucidate the additional roles of this membrane-to-nucleus signal in diverse biological systems. With regard to clinical applications, we expect that our findings will provide a fundamental basis for developing regenerative approaches in various organs.

## Methods

### Reagents and plasmids

The chemical reagents used in this study are as follows: 100 μM nifedipine (Sigma-Aldrich, St. Louis, MO; N7634); 500 μM gadolinium chloride (Sigma-Aldrich, G7532); 10 μM SKF96365 (Sigma-Aldrich, S7809); 1 M EGTA (Sigma-Aldrich, E4378); 500 μM lanthanum chloride (Sigma-Aldrich, L4131); 2 μM ω-Agatoxin IVA (Tocris, Bristol, UK; 2799); 2 μM SNX 482 (Tocris, 2799); 10 μM ω-Conotoxin GVIA (Alomone Labs, C-300); 10 μM U0126 (Sigma-Aldrich, U120); 50 mM potassium chloride (Sigma-Aldrich, P3911); 100 nM AP24534 (Tocris, 4274); 25 μM trifluoperazine dihydrochloride (Sigma Aldrich, T8516).

The following plasmids were purchased from Addgene: pGP-CMV-GCaMP6s was a gift from Douglas Kim (Addgene plasmid # 40753)^33^; pGP-CMV-GCaMP6s-CAAX was a gift from Tobias Meyer (Addgene plasmid # 52228)^34^; AAV-CAG-GFP was a gift from Karel Svoboda (Addgene plasmid # 28014)^35^. The generation procedures for ERK1-dTomato construct were described previously^36^. AAV-CAG-GCaMP6s-CAAX was cloned by replacing the GFP sequence in the AAV-CAG-GFP vector with GCaMP6s-CAAX PCR amplicon flanked BamHI/HindIII restriction sites. pHelper, and pAAV-RC1 plasmids were purchased from Cell Biolabs. RaichuEV-HRas FRET biosensor was kindly gifted from Dr. M. Matsuda (Kyoto University).

### Mouse embryonic organ culture

ICR mice were used for embryonic organ culture. Animal experiment protocol was approved by Seoul National University Institutional Animal Care and Use Committee (approval number: SNU-160322-2). We confirm that all experiments were performed in accordance with relevant guidelines and regulations. Submandibular glands (SMGs) and lungs were harvested from the embryos at E13 (SMG) or at E11.5 (lung) under visualization through a dissecting microscope (Leica, Germany). Organ explants were plated on polycarbonate membranes with 0.1 μm pore size (Watman, Maidstone, UK; 110405), floating on 200 μl DMEM/F12 (Gibco, Grand Island, NY; 21041-025) supplemented with 150 μg/ml ascorbic acid (Sigma-Aldrich, A5960), 50 μg/ml transferrin (Sigma-Aldrich, T8158), and 1% penicillin-streptomycin (Gibco, 15140122) in glass-bottom 50 mm microwell dishes (MatTek Corporation, Ashland, MA; P50G-1.5-14-F). Epithelial separation of SMGs and immersion procedures referred to previous methods^37,38^. Briefly, cultured SMGs were treated with dispase I (0.5 U/ml; Life Technologies, Carlsbad, CA; 17105-041) for 20 min, and washed 3 times with 5% bovine serum albumin (BSA)-DMEM/F12 solution. The mesenchymal parts of SMGs were then removed under a dissecting microscope, and the separated epithelial rudiments were incubated in growth factor-reduced Matrigel (BD Bioscience, San Jose, CA; 356231) diluted with DMEM/F12 culture media [containing ascorbic acid, transferrin, penicillin-streptomycin, 10 ng/ml EGF (R&D System, Minneapolis, MN; 236-EG) and 100 ng/ml Fgf7 (R&D System, 251-KG)] with 1:1 ratio. 20 μl Matrigel were injected into 96 well μ-plates (Ibidi, Munich, Germany; 89646) and incubated at 37 °C for 15 min, and a polycarbonate membrane was placed on the gel. After an additional 15 min, DMEM/F12 culture medium was added.

### Imaging equipment and procedures

SMG morphological evaluation was performed using a digital inverted fluorescence microscope (Nikon, Tokyo, Japan; Ti) equipped with a digital camera (Nikon, DS-Ri2) and a CFI Plan Fluor 4x objective (Nikon) or JuLI Br live cell movie analyzer (NanoEnTek, Seoul, Republic of Korea). Immunofluorescence images were taken by confocal laser scanning microscope (Carl Zeiss, Oberkochen, Germany;LSM700) equipped with Plan-Apochromat 10x, Plan-Apochromat 20x, and C-Apochromat 40x objectives (Carl Zeiss) and with 405, 488, and 555 nm wavelength excitation lasers. Live imaging of epithelial rudiments of SMG and SMG-C6 cells were conducted through a confocal microscope (Carl Zeiss) with a customized live cell chamber (Live Cell Instruments, Seoul, Republic of Korea) that maintained 5% CO_2_ and 37 °C conditions. To visualize peripheral cell movement (Fig. 4I,J), the epithelial rudiments of SMGs were briefly stained with 1 μg/ml Hoechst 33342 (Thermo Fisher Scientific, Waltham, MA; H3570) –culture media solution for 1 h. After staining, cells were washed with culture medium two times.

### Adeno-associated virus (AAV) production and transduction

AAVs were produced and purified with a simplified polyethylene glycol (PEG)-based method^39^. For AAV plasmid transfection, human embryonic kidney (HEK)-293T cells were prepared with 70~80% confluence in Dulbecco’s modified Eagle’s medium (DMEM; WelGene, Daegu, Republic of Korea; LM-001-05) containing 10% fetal bovine serum (FBS). Lipofection was conducted using Lipofectamine 2000. AAV plasmids–AAV-CAG-GCaMP6s-CAAX, pHelper, and pAAV-RC1 were transfected at a 1:1:1 ratio. After 48 h, the transfected cells were detached by brief treatment of 0.5 M EDTA solution (pH 8), and collected by centrifugation at 1000 rpm for 10 min. The cell pellets were resuspended in phosphate buffered saline (PBS) and induced to release viral particles by repeated freeze-thaw cycles between −80 °C (deep freezer) and 37 °C (water bath). After centrifugation (13200 rpm, 10 min), the supernatants were mixed with 40% polyethylene glycol (Sigma-Aldrich, 89510) solution with 2.5 N NaCl at a 1:4 ratio. The mixture was incubated at 4 °C for 1 h, then centrifuged at 2000 rpm for 30 min. The supernatants were replaced with HEPES buffer-chloroform 1:1 solution, followed by vortexing (2 min) and centrifugation (400 rpm, 5 min). The upper solution in separated layers was collected and the chloroform was allowed to evaporate for 30 min. The collected AAV solution was dialyzed by two steps with sequential use of dialysis tubes with different pore sizes (3 KDa and 50 KDa nominal molecular weight limit; Millipore, UFC8003 and 4310). Dialyzed AAVs (1 × 10^11–12^ copies/ml) were diluted in DMEM/F12 containing 1% penicillin-streptomycin. Epithelial rudiments of SMGs were incubated in the viral media for 1 h at room temperature. The rudiments were washed two times with DMEM/F12 containing 1% penicillin-streptomycin, and incubated in Matrigel.

### Rat submandibular epithelial cell line (SMG-C6) culture and transfection

SMG-C6 cells were kindly gifted from Prof. Guang-Yan Yu (Peking Univ.). SMG-C6 cells were cultured in 5% CO_2_ at 37 °C with DMEM/F12 (Sigma-Aldrich, D8900) containing 2.5% FBS, 5 μg/ml transferrin, 1.1 μM hydrocortisone, 100 nM retinoic acid, 2 nM T3, 5 μg/ml insulin, 80 ng/ml EGF, 5 mM glutamine, 50 μg/ml gentamicin sulfate, and 1% penicillin-streptomycin. For plasmid transfection, the cells were plated on glass-bottom 96-well plates (Matrical Bioscience, Spokane, WA) with 2 × 10^4^ cells/well density, then cultured for 24 h. Transfection was conducted using Lipofectamine 2000 (Invitrogen, 11668) according to the manufacturer’s instructions. Serum starvation was performed with serum-deprived culture media at least 4 h before experiments.

### Immunofluorescence

SMG cultures were fixed by 4% formaldehyde treatment for 20 min at room temperature, and washed 3 times with PBS containing 1% Tween-20 (PBST) for 10 min. The cultures were permeabilized with PBS containing Triton X-100 (PBSX) for 20 min at room temperature and washed three times with PBST. PBS containing 0.1% BSA and 10% FBS was used as a blocking solution. After 1 h, the blocking solution was replaced with primary antibodies in PBST (1:200) and incubated on laboratory shaker at 4 °C. The primary antibody incubation was followed by washing 3 times and the cultures were incubated with secondary antibody-PBST solution (1:500) at least 6 h at room temperature. The antibodies used in immunofluorescence were as follows: mouse monoclonal anti-p-Tyr (Santa Cruz Biotechnology, Santa Cruz, CA; sc-508); mouse monoclonal anti-dihydropyridine receptor alpha-1 (Thermo Fisher Scientific, MA3–920); rabbit polyclonal anti-Ca_V_1.2 (Alomone Labs, Jerusalem, Israel; ACC-003); rabbit polyclonal anti-Ca_V_1.3 (Alomone Labs, ACC-005); mouse monoclonal anti-E-cadherin (Santa Cruz Biotechnology, sc-8426); rabbit polyclonal E-cadherin (Santa Cruz Biotechnology, sc-7870); mouse monoclonal anti-γ-tubulin (Sigma Aldrich, T6557); rabbit polyclonal anti-phospho-p44/42 MAPK (Cell Signaling Technology, Beverly, MA; 9101); mouse monoclonal anti-phospho-Histone H3 (Cell Signaling Technology, 9706); mouse monoclonal anti-actin, α-smooth muscle (Sigma Aldrich, A5228); goat anti-mouse IgG (H + L), Alexa Fluor® 488 conjugate (Thermo Fisher Scientific, a11001); goat anti-rabbit IgG (H + L), Alexa Fluor® 594 conjugate (Thermo Fisher Scientific, R37117).

### PCR

Total RNA of SMG tissues and cells was extracted by RNeasy Mini Kit (Qiagen, Hilden, Germany; 74140). 1 μg of total RNA was used for synthesizing cDNA through reverse transcriptase (SuperScript III First-Strand Synthesis System; Thermo Fischer Scientific, 18080-051) with oligo-dT and random hexamer primers. Nested PCR (Supplementary Fig. S1E) was conducted using Platinum Taq DNA Polymerase (Thermo Fischer Scientific, 10966-018). Real-time PCR was performed using SYBR PCR master mix (Applied Biosystems, Foster City, CA; 4309155) with a real-time PCR instrument (Applied Biosystems, 7200). The sequences of primers were as follows (5′ to 3′)^40^:

Ca_V_1.1-forward: GTTACATGAGCTGGATCACACAG; Ca_V_1.1-reverse: ATGAGCATTTCGA-TGGTGAAG; Ca_V_1.2- forward: CATCACCAACTTCGACAACTTC; Ca_V_1.2- reverse: CAGG-TAGCCTTTGAGATCTTCTTC; Ca_V_1.3- forward: ACATTCTGAACATGGTCTTCACAG; Ca_V_1.3- reverse: AGGACTTGATGAAGGTCCACAG; Ca_V_1.4-forward: CTCTTCATCTGTG-GCAACTACATC; Ca_V_1.4- reverse: GTACCACCTTCTCCTTGGGTACTA; αSMA-outer forward: GAAGAGGAAGACAGCACAGC; αSMA-outer reverse: AGAGGCATAGAGGGAC-AGCA; αSMA-inner forward: GGCTCTGGGCTCTGTAAGG; αSMA-inner reverse: CTCTTG-CTCTGGGCTTCATC; GAPDH-outer forward: ACTTGAAGGGTGGAGCCAAA; GAPDH-outer reverse: TTCAGCTCTGGGATGACCTT; GAPDH-inner forward: TCCTGCACCACCA-ACTGCTT; GAPDH-inner reverse: TGGCAGTGATGGCATGGAC.

### Fluorescence *in situ* hybridization (FISH)

Custom Stellaris® FISH Probes were designed against Cacna1s (NM_014193.2) and Cacna1c (NM_009781.4) by utilizing the Stellaris® RNA FISH Probe Designer (Biosearch Technologies, Novato, CA; available online at www.biosearchtech.com/stellarisdesigner). Samples were hybridized with the customized RNA FISH Probe set labeled with Fluorescein Dye (Biosearch Technologies, Inc.), following the manufacturer’s instructions (available online at www.biosearchtech.com/stellarisprotocols).

### Immunoblotting

SMG-C6 cells were lysed in ice-cold RIPA buffer (GenDEPOT, Barker, TX; R4200-010) and protein concentrations were measured using s spectrophotometer (Nanodrop; Thermo Fischer Scientific, ND-1000). Protein samples were separated using 10% SDS-PAGE gels (Bio-Rad, Hercules, CA). After electrophoresis in a Power-Pac Basic system (Bio-Rad), proteins were transferred to nitrocellulose membranes using an iBLOT 2 Dry Blotting system (Thermo Fisher Scientific, IB21001). The membranes were blocked with 10% non-fat milk and incubated with anti-ERK antibodies (1:1000; Cell Signaling Technology, 9102) and anti-pERK antibodies (1:1000; Cell signaling, 9101) at 4 °C overnight. After washing, membranes were incubated with anti-rabbit IgG-HRP (1:5000; Santa Cruz Biotechnology, sc-2030). Immunoreactivity was visualized by ECL reagents (Thermo Fisher Scientific, 32106) and detected by the Chemidoc XRS+ system (Bio-Rad Laboratories).

### Data analysis

Images were analyzed using Fiji software (National Institutes of Health). Bud numbers of SMG cultures were manually counted based on phase contrast images. To measure VDCC expression (Fig. 2F), we calculated the average intensity of inmmunolabeled VDCC signals on epithelial membrane of whole eSMG culture. Cell movement in the peripheral layer of SMGs (Fig. 4J) was recorded by manual tracking based on confocal fluorescent images. To identify mitotic cells (Fig. 4B,F and G), we selected the cells showing centrally-arranged and condensed DAPI signals between two separated mitotic centers represented by condensed γ-tubulin signals (Fig. 4E and Supplementary Fig. S4A). The mitotic angle (θ) was calculated from parameters in Z-stack images (step width: 1 µm) of mitotic cells taken by a confocal microscope (Carl Zeiss). The equation is as follows:

1 $$\theta =\arcsin \frac{{\rm{c}}}{\sqrt{{{\rm{a}}}^{2}+{{\rm{b}}}^{2}}}$$

a: Z-stack distance between two γ-tubulin signals; b: horizontal distance between two γ-tubulin signals when the signals were orthogonally projected to a single virtual plane; a^2^ + b^2^: actual distance between two γ-tubulin signals; c: difference between distances of each γ-tubulin signal-to-acinar surface.

### Statistical analysis

All experiments were performed at least two times for biological replicates. The difference between the two groups was determined using a two-tailed t-test. Multiple significance tests were performed using one-way ANOVA, and post hoc analysis was conducted via two-tailed t-test with Bonferroni correction. The Analysis ToolPak-VBA in Excel was used for all statistical analyses.

### Data availability statement

All data from the current study that were generated or analyzed are available upon reasonable request from the corresponding author. All data needed to evaluate the conclusions in the paper are present in the paper and/or the Supplementary Materials. Additional data related to this paper may be requested from the authors.

## Electronic supplementary material

Supplementary information

Video1

Video2

Video3

Video4
