# Supplementary material for: Voltage-dependent Ca2+ channels promote branching morphogenesis of salivary glands by patterning differential growth
Source: Sci Rep. 2018 May 15;8:7566. doi: 10.1038/s41598-018-25957-w (PMC5954160; doi:10.1038/s41598-018-25957-w)
Supplement: Supplementary file 1 — Supplementary information [file 41598_2018_25957_MOESM1_ESM.doc]

**Supplementary information**

**Voltage-dependent Ca^2+^ channels promote branching morphogenesis of salivary glands by patterning differential growth**

J. M. Kim,^1^ S. Choi,^2^ S.W. Lee,^2^ K. Park^2^,*

**Affiliations**

^1^ Department of Dentistry, CHA Bundang Medical Center, CHA University, Seongnam, 13496, South Korea.

^2^ Department of Physiology, School of Dentistry, Seoul National University and Dental Research Institute, Seoul, 03080, South Korea.

*Correspondence to: kppark@snu.ac.kr


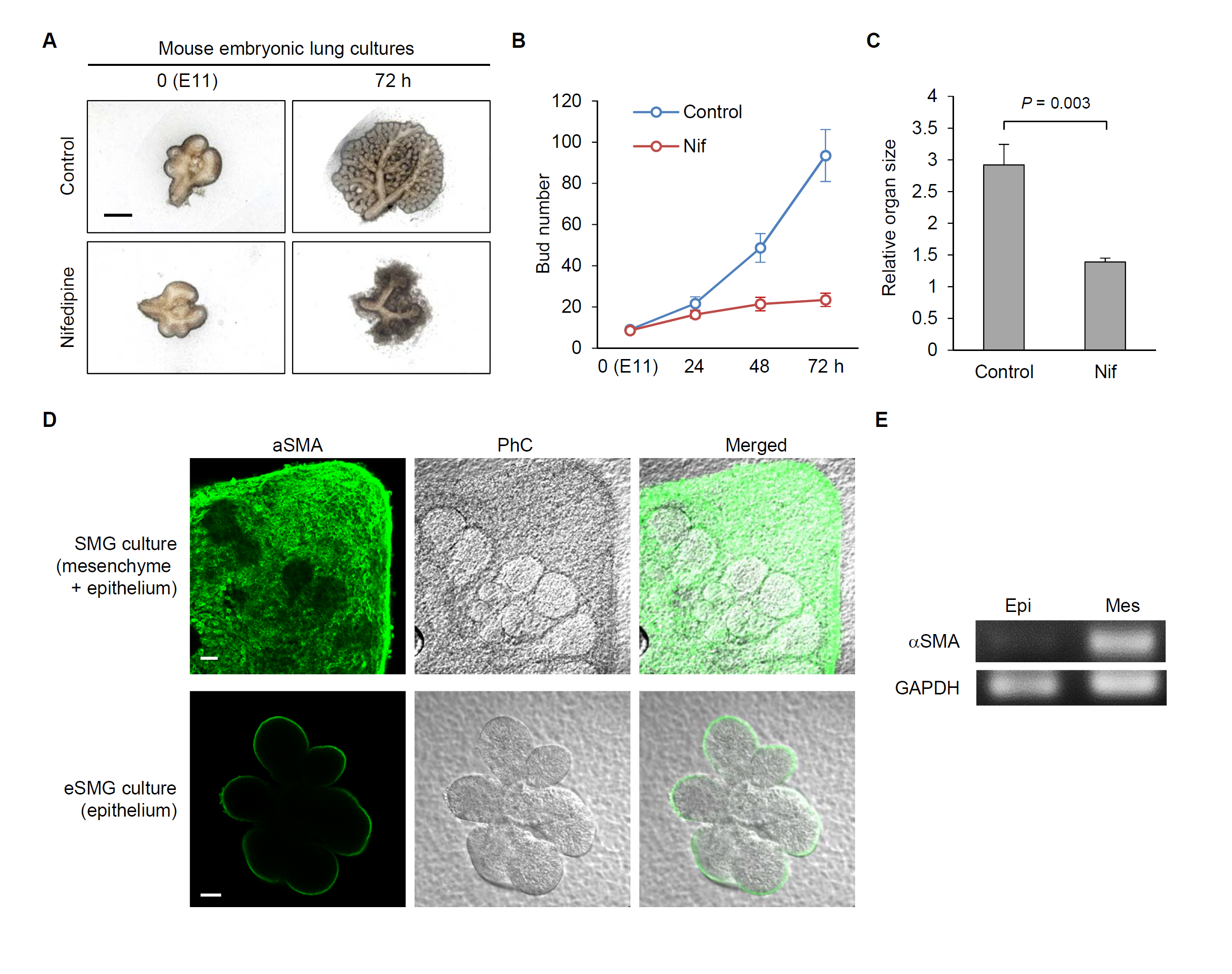


**Fig. S1. The effect of L-type VDCC on mouse embryonic organ development.**

(**A**) Morphological changes of embryonic lung cultures upon 100 μM nifedipine treatment (E11-14). (**B** and **C**) Bud numbers (B) and organ size (C) of embryonic lung cultures upon 100 μM nifedipine treatment. n=7. Data are represented as mean ± SEM. (**D**) Immunostaining images of αSMA (green) in SMG (upper) and eSMG (lower) cultures.PhC: phase contrast image. (**E**) mRNA expression of αSMA in the epithelial (Epi) and mesenchymal (Mes) cells of SMG cultures. Scale bars: 50 (A) 500 (C) μm


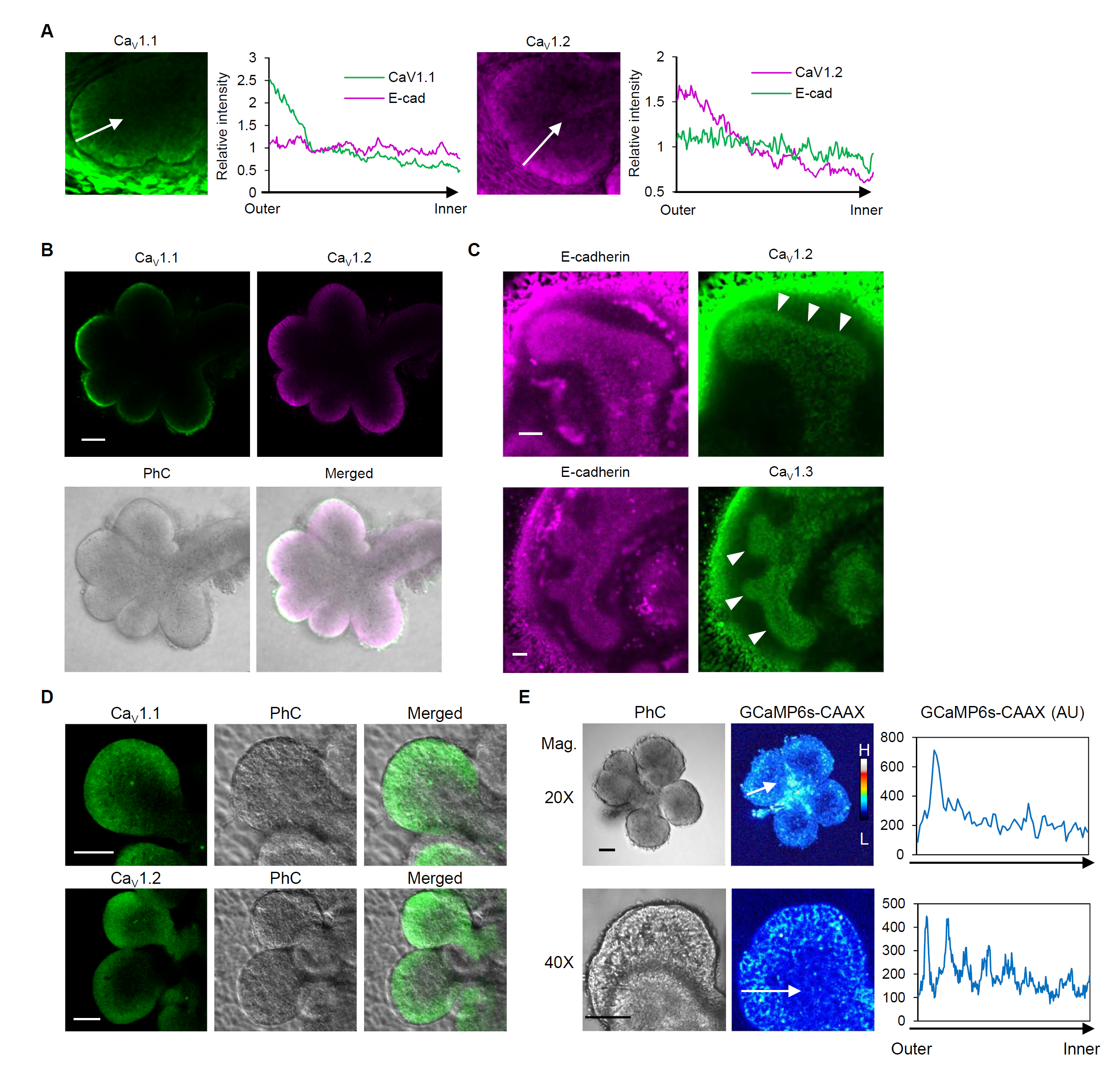


**Fig. S2. Functional expression of L-type VDCCs in developing organs.**

(**A**) Line-scan intensities of immunolabeled Ca_V_1.1 and 1.2 channels in developing buds. White arrows indicate the line-scanned region. (**B**) Expression pattern of Ca_V_1.1 and 1.2 channels in epithelial rudiments of SMG (eSMG). PhC: phase contrast image. (**C**) Immunostaining images of Ca_V_1.2 and 1.3 channels (green) and E-cadherin (magenta) in embryonic lung cultures, focused on the peripheral plane of developing tubes. Arrow heads indicate the border of epithelial tubes. (**D**) Images of fluorescent *in situ* hybridization labeling mRNA of Ca_V_1.1 and 1.2 channels in eSMG cultures. (**E**) Left: Images of membrane-tethered Ca^2+^ biosensor (GCaMP6s-CAAX) expressing eSMGs taken with different magnification. Arrows indicate the line-scan region. Right: quantified graphs displaying GCaMP6s-CAXX intensities in the line-scan region. Scale bars: 50 μm


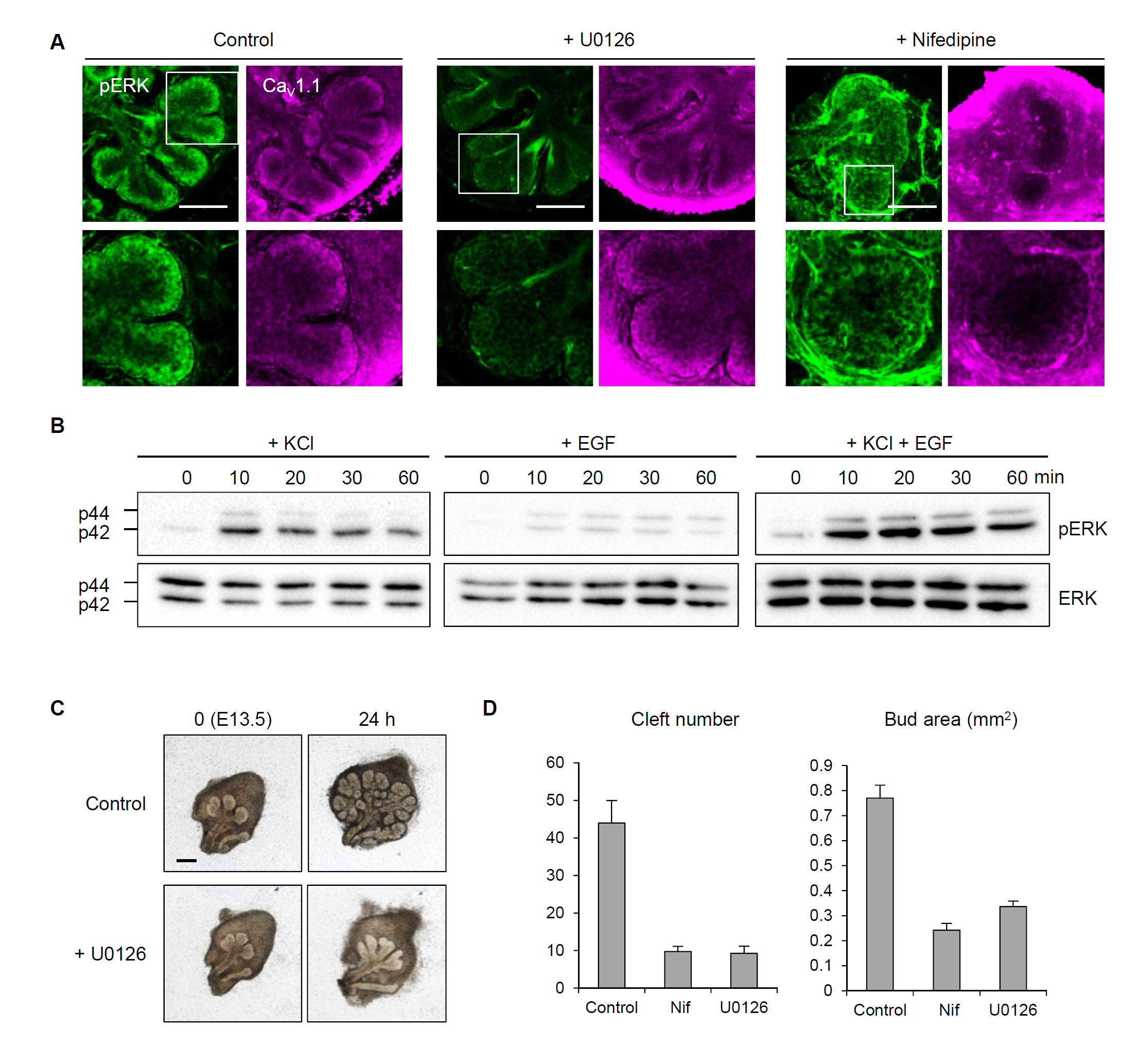


**Fig. S3. Signaling hierarchy and synergism between ERK and VDCC activity.**

(**A**) Immunostaining images of phosphorylated ERK (green) and Ca_V_1.1 channels (magenta) in SMG cultures upon U0126 and nifedipine treatment. (**B**) Immunoblotting results of phosphorylated ERK (upper bands) and total ERK (lower bands) after KCl and/or EGF treatment. The grouping of blots were cropped from different different gels under same exposure condition. (**C**) Morphological changes of SMG cultures upon U0126 treatment. (**D**) Cleft numbers (left) and total bud area (right) of SMG cultures upon nifedipine (Nif) and U0126 treatment. n=4. Data are represented as mean ± SEM. Scale bars: 200 μm


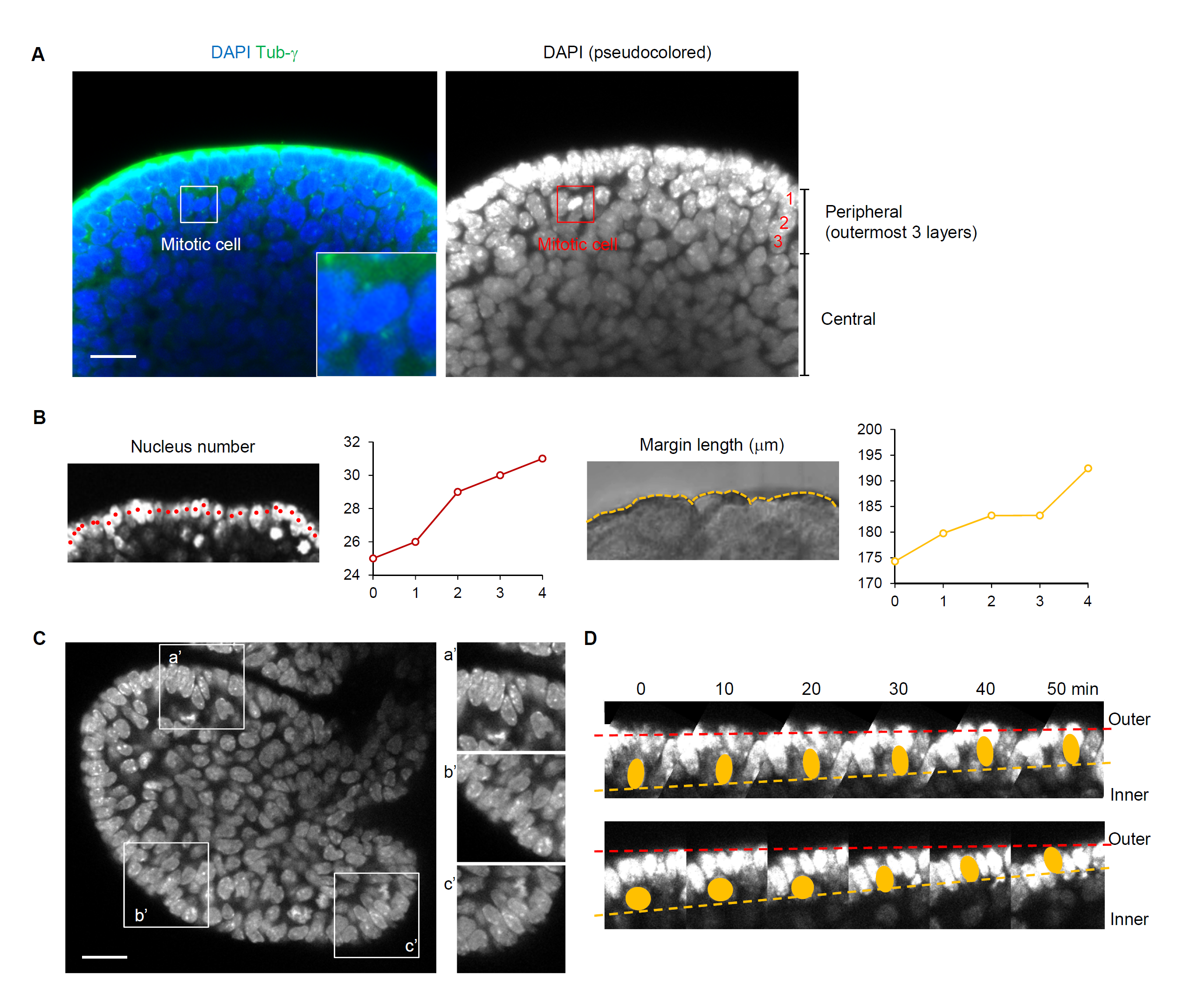


**Fig. S4. Intraacinar cell dynamics in the peripheral layer.**

(**A**) Representative images of mitosis distribution in developing eSMGs. Boxed regions indicate the enlarged image of mitotic cell. (**B**) Quantitative results of nucleus number (left) and margin length (right) of the peripheral epithelium in developing eSMGs. (**C**) Fixed image of eSMG culture stained with DAPI. White boxes indicate pseudostratified-like epithelial regions. (**D**) Serial images of the nucleus of acinar cells (yellow ellipses) migrating toward the peripheral layer. Dotted red lines indicate the external border of epithelial buds. Dotted yellow lines indicate the posterior border of the nucleus in migrating cells. Scale bars: 20 μm


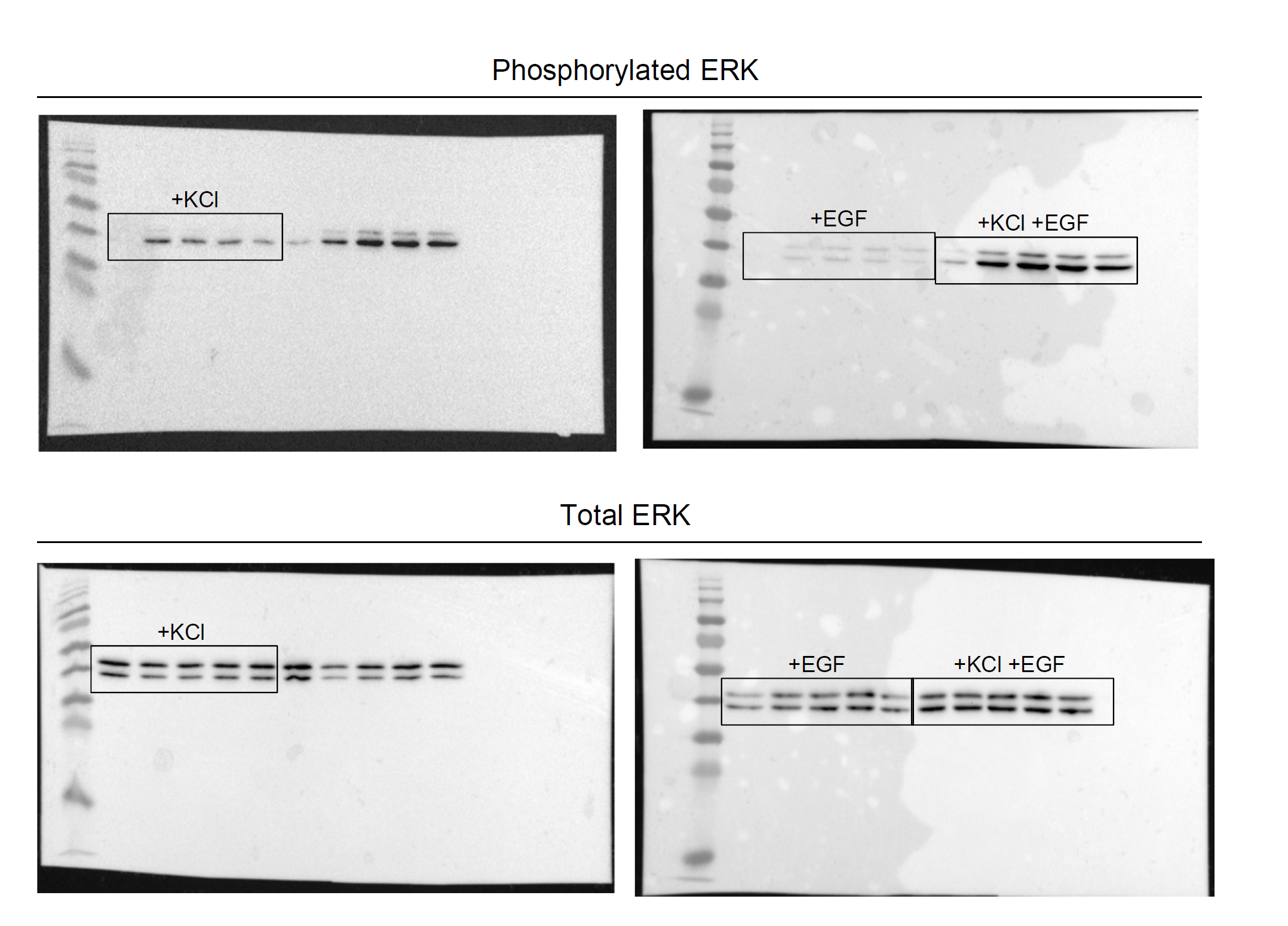


**Fig. S5. Full-length blots of cropped images in Fig. S3B.**

**Supplementary Video Legends**

**Supplementary Video 1. The effect of L-type VDCCs in SMG branching morphogenesis.**

The branching process of the untreated control (left) and nifedipine-treated (right) SMG cultures (E13.5). Timelapse phase contrast images were taken for 18 h with 10 min intervals. Scale bar: 100 μm

**Supplementary Video 2. L-type VDCC-induced ERK translocation in SMG-C6 cells.**

Pseudocolored timelapse movie of GCaMP6s-CAAX (left) and ERK-dTomato (right) signals in SMG-C6 cells upon KCl treatment. Images were taken for 35 min with 30 s intervals. Scale bar: 20 μm

**Supplementary Video 3. Epithelial inclusion of developing eSMGs.**

Pseudocolored 3D reconstruction images of developing eSMGs at E13 (left) and E13.5 (right). The nucleus of epithelial cells in the peripheral layer was labeled by Hoechst dye (see supporting online material). Scale bar: 20 μm

**Supplementary Video 4. Epithelial cell dynamics at the cleft initiation site.**

Pseudocolored and phase contrast movies displaying the cleft initiation process. The nucleus of epithelial cells in the peripheral layer was labeled with Hoechst dye. Images were taken for 4 h with 10 min intervals. Scale bar: 20 μm
